# The Prognostic Value of Cardiac Biomarkers and Echocardiography in Critical COVID-19

**DOI:** 10.3389/fcvm.2021.752237

**Published:** 2021-11-05

**Authors:** Bert Zwaenepoel, Sebastiaan Dhont, Eric Hoste, Sofie Gevaert, Hannah Schaubroeck

**Affiliations:** ^1^Department of Cardiology, Ghent University Hospital, Ghent University, Ghent, Belgium; ^2^Department of Intensive Care Medicine, Ghent University Hospital, Ghent University, Ghent, Belgium; ^3^Research Foundation – Flanders (FWO), Brussels, Belgium

**Keywords:** COVID-19, hs-cTnT, NT-proBNP, ICU, myocardial injury, myocardial biomarker

## Abstract

**Background:** Early risk stratification is crucial in critically ill COVID-19 patients. Myocardial injury is associated with worse outcome. This study aimed to evaluate cardiac biomarkers and echocardiographic findings in critically ill COVID-19 patients and to assess their association with 30-day mortality in comparison to other biomarkers, risk factors and clinical severity scores.

**Methods:** Prospective, single-center, cohort study in patients with PCR-confirmed, critical COVID-19. Laboratory assessment included high sensitive troponin T (hs-cTnT) and N-terminal pro-brain natriuretic peptide (NT-proBNP) on admission to ICU: a hs-cTnT ≥ 14 pg/mL and a NT-proBNP ≥ 450 pg/mL were considered as elevated. Transthoracic echocardiographic evaluation was performed within the first 48 h of ICU admission. The primary outcome was 30-day all-cause mortality. Predictive markers for mortality were assessed by ROC analysis and cut-off values by the Youden Index.

**Results:** A total of 100 patients were included. The median age was 63.5 years, the population was predominantly male (66%). At the time of ICU admission, 47% of patients had elevated hs-cTnT and 39% had elevated NT-proBNP. Left ventricular ejection fraction was below 50% in 19.1%. Elevated cardiac biomarkers (hs-cTnT *P*-value < 0.001, NT-proBNP *P*-value = 0.001) and impaired left ventricular function (*P*-value = 0.011) were significantly associated with mortality, while other biomarkers (D-dimer, ferritin, C-reactive protein) and clinical scores (SOFA) did not differ significantly between survivors and non-survivors. An optimal cut-off value to predict increased risk for 30-day all-cause mortality was 16.5 pg/mL for hs-cTnT (OR 8.5, 95% CI: 2.9, 25.0) and 415.5 pg/ml for NT-proBNP (OR 5.1, 95% CI: 1.8, 14.7).

**Conclusion:** Myocardial injury in COVID-19 is common. Early detection of elevated hs-cTnT and NT-proBNP are predictive for 30-day mortality in patients with critical COVID-19. These markers outperform other routinely used biomarkers, as well as clinical indices of disease severity in ICU. The additive value of routine transthoracic echocardiography is disputable and should only be considered if it is likely to impact therapeutic management.

## Introduction

Currently, severe acute respiratory syndrome coronavirus 2 (SARS-CoV-2) has infected almost 230 million people, resulting in more than 4.6 million registered deaths (1). Based upon the severity of illness, the National Institutes of Health (NIH) proposes five categories of Coronavirus Disease 2019 (COVID-19): asymptomatic, mild, moderate, severe and critical. The latter contains individuals with respiratory failure, septic shock, and/or multiple organ dysfunction requiring intensive care (2).

Myocardial injury is defined by an elevation of cardiac troponin. In case of myocardial infarction, this elevation occurs in combination with clinical features of ischemia, e.g., electrocardiographic changes, ischemic symptoms or imaging of new loss of viable myocardium or new regional wall motion abnormalities (3). Myocardial injury is common in COVID-19. The presence of values above the upper reference limit (URL) for high sensitive troponin (hs-cTnT) in COVID-19 patients varies widely, ranging from 20% in cohorts of hospitalized patients to more than 50% in critically ill patients (4–7). Ischemic or non-ischemic causes can mediate myocardial injury. Ischemic cardiac damage can be subdivided in type 1 and type 2 ischemia. The underlying pathophysiology of type 1 ischemia in COVID-19 is not fully understood. On one hand, the inflammatory response due to a COVID-19 infection may lead to plaque instability by activating inflammatory cells and release of inflammatory mediators, causing oxidative stress. On the other hand, COVID-19 infection is associated with endothelialitis and a prothrombotic state (8–11). Type 2 ischemia can be attributed to several factors such as hypoxemia, vasopressor use and suboptimal fluid balance, leading to a demand-supply inequity of oxygen (10). Non-ischemic injury may find its origin in various mechanisms such as myocarditis, Takotsubo syndrome, arrythmias, pulmonary embolism, and septic shock (12–16).

Data about natriuretic peptides are more scarce, though up to 48% of critical COVID-19 patients present with elevated levels of N-terminal pro-brain natriuretic peptide (NT-proBNP), reflecting hemodynamic stress (17). The presence of circulating NT-pro-BNP in patients with critical COVID-19 can be attributed to several factors. Myocardial injury may lead to cardiac dysfunction and increased ventricular wall stress, which can be increased further by the use of mechanical ventilation and vasopressor agents (18). Hypoxia-induced pulmonary hypertension may further aggravate myocardial wall stress by increasing right ventricular afterload (19, 20).

Echocardiographic abnormalities, such as left ventricular systolic and diastolic dysfunction as well as right ventricular impairment, are observed in up to half of all COVID-19 patients undergoing echocardiography (21–24).

Elevated cardiac biomarkers and echocardiographic abnormalities, especially reduced ventricular contractility, are associated with worse clinical outcome including mortality in COVID-19 patients (10, 17, 18, 23, 25–27). As most of the published reports are retrospective studies, the current role of cardiac biomarkers and/or echocardiography in the prognostication of COVID-19 patients is still unclear. Different cardiac societies therefore have recommended against the routine use of these parameters for prognostic purposes (16, 28).

The purpose of this study was to prospectively evaluate the presence of elevated cardiac biomarkers and echocardiographic abnormalities in critical COVID-19 patients at the time of admission to the intensive care unit (ICU), to assess their association with 30-day all-cause mortality and to compare their prognostic performance to that of other biomarkers, risk scores and risk factors.

## Methods

### Study Design, Data Collection, and Study Outcome

This prospective, single-center, cohort study was carried out at the ICU of the Ghent University Hospital in Belgium, a 1.061-beds tertiary care center, between April 2020 and April 2021. The study was approved by the local ethical committee (BC-07568, April 1st, 2020). Inclusion criteria were: age 18 years or older, inclusion within 48 h of ICU admission, severe COVID-19 as diagnosed by real-time reverse-transcriptase polymerase chain reaction assays, and informed consent of the patient or legal representative. At the time the study protocol was made and the study was started, there were no epidemiological data available about cardiac biomarkers and echocardiography in critical COVID-19. The necessary number of patients to demonstrate differences between survivors and non-survivors could therefore not be estimated and therefore no power analysis was made. It was decided to include all consecutive patients admitted to our ICU in the first and second COVID-19 wave which arose in Belgium.

Demographics, pre-existing comorbidities, chronic medication, administered medication on ICU, clinical risk-scores and ratios (total and respiratory sequential organ failure assessment score (SOFA) and PaO2/FiO2-ratio (P/F ratio) on admission were automatically abstracted from the electronic health record on the moment of admission. Laboratory assessment included hs-cTnT (electrochemiluminescence immunoassay (ECLIA), Roche Cobas 8000 e80, Roche Diagnostics, Switzerland), NT-proBNP (ECLIA, Roche Cobas 8000 e80, Roche Diagnostics, Switzerland), C-reactive protein (CRP) (photometric measurement, Architect c16000, Abbott, Abbott Laboratories, Illinois, United States), ferritin (chemiluminescent Microparticle Immunoassay (CMIA), Architect i2000SR, Abbott, Abbott Laboratories, Illinois, United States) and D-dimer (immunoturbidimetry, STA R Max2, Stago, France). The first value upon admission was withheld when several blood samples were taken within 1 day. The cut-off for hs-cTnT was 14 pg/ml (corresponding with levels above the 99th percentile of a normal reference population) and for NT-proBNP 450 pg/mL. Patients were continuously monitored with a 3-lead electrocardiogram (ECG) and an additional 12-lead ECG was obtained on a daily base.

During follow-up, the use of vasopressors, mechanical ventilation and/or venovenous extracorporeal membrane oxygenation was recorded. Bedside transthoracic echocardiography was performed within the first 48 h of inclusion, using a portable ultrasound machine CX50 (Philips Medical Systems, Andover, MA). The left ventricular ejection fraction (LVEF) was estimated with eyeball-method (normal, midrange and reduced) because of two reasons. First, acquisition of good quality images is often hard to obtain in critically ill patients and therefore more sophisticated methods (e.g., Simpson biplane, speckle tracing) to estimate the LVEF are not always feasible. Second, visually estimated ejection fraction has already shown to be extremely effective, rapid and consistent with quantitative echocardiographic assessment and is therefore a feasible method in critically ill patients (29). Other parameters that were obtained are: LV end diastolic diameter (LVEDD), diastolic function (E/A ratio and E/e′ septal), tricuspid annular plane systolic excursion (TAPSE), estimate systolic pulmonary arterial pressure (SPAP) using the maximal tricuspid regurgitation velocity with CW Doppler, valvular function and presence of pericardial fluid. Diastolic function was dichotomized according to indices of diastolic dysfunction and increased left ventricular pressure (E/A > 1.5 and/or E/e' septal > 14). Echocardiography was performed by six skilled sonographers, all images were stored in the Picture Archiving and Communication System (PACS) of the hospital. The primary outcome of the study was all-cause 30-day mortality.

### Data Analysis

The statistical analysis was performed using SPSS statistics (Version 27.0, IBM Corp, Armonk, NY). Normality of the distribution of continuous variables was tested by the Shapiro Wilk test. Categorical variables are shown as frequencies, and continuous variables as mean (standard deviation) or median (interquartile range) based upon normality of distribution. Comparison of categorical variables was performed using Chi-squared tests and for comparison of continuous variables Mann-Whitney U tests was used. Predictive markers for mortality were assessed by receiver operating characteristic (ROC) analysis and cut-off values by the Youden Index. The latter is a frequently used summary measure of the ROC curve. It represents the effectiveness of a diagnostic marker and enables the selection of an optimal threshold value (30). Multivariate logistic regression analysis was conducted to investigate risk factors for mortality. Since the number of events of the independent variable 30-d mortality was 21, the number of covariates that were added into the regression analysis to explore adjusted OR's was limited to 1. All tests were 2-sided with *P* < 0.05 considered statistically significant.

## Results

### Patient Characteristics, Comorbidities, Chronic Medication, and Outcomes

**Figure 1:**
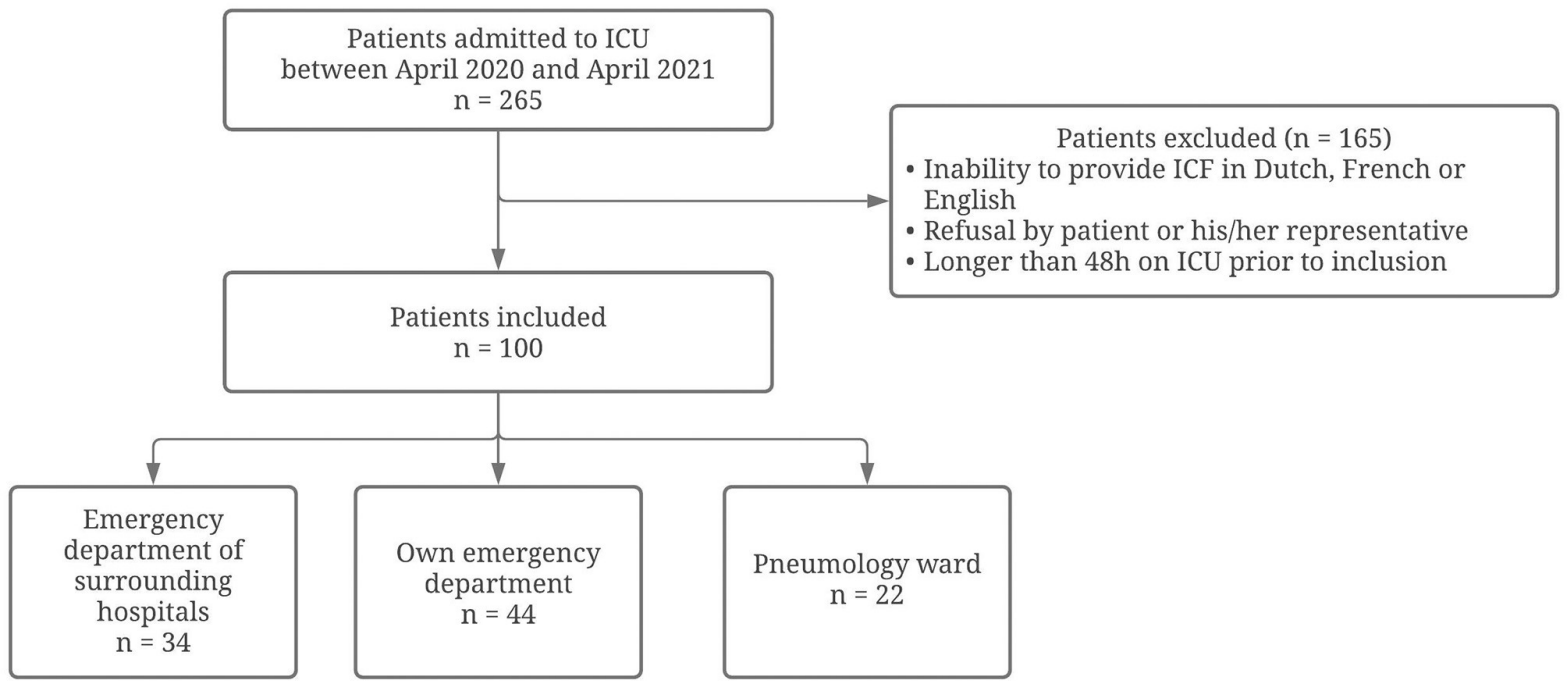
Flow diagram on inclusion and exclusion of patients in the current study. Flow diagram reporting the numbers of included and excluded patients in the current study.

In the study period, a total of 265 critically ill COVID-19 patients were admitted to our ICU department. The main reasons for exclusion were: more than 48 h on ICU prior to inclusion, inability to provide informed consent in Dutch, French or English (as the informed consent forms were only in these languages available) and refusal to participate by the patient or his/her representative (*n* = 165). A total sample of 100 critically ill COVID-19 patients was finally included within 48 h of ICU admission. Included patients originated from emergency departments of surrounding hospitals (*n* = 34), our own emergency department (*n* = 44) or the pneumology ward (*n* = 22). A flow diagram to illustrate the patients' selection can be found in Figure 1.

Baseline characteristics are presented in Table 1. Median age was 63.5 years, and the population was predominantly male (66%). None of the included patients were vaccinated against SARS-CoV-2. The mean body mass index (BMI) was 28.7 kg/m^2^. Following comorbidities were recorded upon admission: arterial hypertension (42%), diabetes mellitus (28%), sleep apnea (6%), chronic obstructive pulmonary disease (COPD)/asthma (8%), coronary artery disease (17%), peripheral artery disease (6%), hypercholesterolemia (40%) and kidney disease upon admission (estimated glomerular filtration rate <60 mL/min in 20% of patients). Chronic medication consisted of statins (37%), antidiabetic drugs (metformin 24%, SGLT2 inhibitors 2%, others 15%), antihypertensive drugs (ACE-inhibitors 28%, beta blockers 40%, others 25%) and antithrombotic/anticoagulant drugs (aspirin 20%, P2Y12 inhibitors 4%, NOAC/VKA 12%). During admission on ICU patients received additional treatment with dexamethasone (74%), remdesivir (12%), hydroxychloroquine (11%) and convalescent plasma (10%). During admission 54% of patients required vasopressors, 60% of patients were mechanically ventilated and 7% of patients were supported with venovenous extracorporeal membrane oxygenation. On admission the median total SOFA-score was 3.0, with a respiratory SOFA-score of 2.0. The median P/F-ratio on admission was 96.3 mmHg. The median length of stay in ICU was 10 days. Within the first 30 days after inclusion 21 patients died (21%). Non-survivors were significantly older, were more often male and more often had kidney disease, sleep apnea, COPD/asthma and hypercholesterolemia. Respiratory SOFA, total SOFA and P/F ratio did not differ significantly between survivors and non-survivors.

**Table 1:** Baseline demographics, disease severity, laboratory assessments, and echocardiographic parameters of patients on admission to the intensive care unit.

**Demographics (*****n*** **=** **100)**	
Age (y)	63.5 (IQR 57.0–71.0)
Gender	
Male	66 (66.0 %)
Female	34 (34.0 %)
BMI (kg/m^2^)	28.7 (IQR 25.1–33.6)
Smoking history	
Never smoker	57 (57.0%)
Former smoker	36 (36.0%)
Active smoker	7 (7.0%)
Transferred from	
Emergency department	44 (44.0%)
Pneumology ward	22 (22.0%)
Surrounding hospital (not ICU)	34 (34.0%)
**Comorbidities (*****n*** **=** **100)**	
Arterial hypertension	42 (42.0%)
Kidney disease upon admission	
eGFR <30 mL/min	3 (3.0%)
eGFR 30–60 mL/min	17 (17.0%)
eGFR > 60 mL/min	80 (80.0%)
Diabetes mellitus type 2	28 (28.0%)
Obstructive sleep apnea	6 (6.0%)
Chronic obstructive pulmonary disease / asthma	8 (8.0%)
Hypercholesterolemia	40 (40.0%)
Coronary artery disease	17 (17.0%)
Peripheral artery disease	6 (6.0%)
**Chronic medication (*****n*** **=** **100)**	
Use of statins	37 (37.0%)
Use of antidiabetic drugs	
Metformin	24 (24.0%)
SGLT2 inhibitor	2 (2.0%)
Other	15 (15.0%)
Use of antihypertensive drugs	
ACE-inhibitor	28 (28.0%)
Beta blocker	40 (40.0%)
Other	25 (25.0%)
Use of antithrombotic / anticoagulant drugs	
Aspirin	20 (20.0%)
P_2_Y_12_ inhibitor	4 (4.0%)
NOAC or VKA	12 (12.0%)
**Medication administered on ICU (*****n*** **=** **100)**	
Dexamethasone	74 (74.0%)
Remdesivir	12 (12.0%)
Hydroxychlorquine	11 (11.0%)
Convalescent plasma	10 (10.0%)
**Severity of illness (*****n*** **=** **100)**	
Total SOFA-score on admission	3.0 (IQR 2.0–8.0)
Respiratory SOFA-score on admission	2.0 (IQR 2.0–3.0)
P/F ratio (IQR) on admission	96.3 (IQR 71.6–124.7)
Use of vasopressors during admission	54 (54.0 %)
Use of mechanical ventilation during admission	60 (60.0 %)
Use of vv-ECMO during admission	7 (7.0 %)
**Inflammatory markers at time of inclusion (*****n*** **=** **100)**	
CRP (mg/L)	136.5 (IQR 67.0–201.3)
D-dimer (ng/mL)	1020.0 (IQR 660.0–1795.0)
Ferritin (μg/L)	1139.0 (IQR 640.8–2346.8)
**Cardiac biomarkers at time of inclusion (*****n*** **=** **100)**	
hs-cTnT (μg/L)	
≥14 μg/L	47 (47.0 %)
<14 μg/L	53 (53.0 %)
NT-proBNP (pg/mL)	
≥450 pg/mL	39 (39.0 %)
<450 pg/mL	61 (61.0 %)
**Echocardiography parameters at time of inclusion**	
LVEF (%) (***n*** = 89)	
Normal (>50%)	72 (80.9 %)
Midrange (40–50%)	16 (18.0 %)
Reduced (<40%)	1 (1.1 %)
LVEDD (mm) (*n* = 83)	46.0 (IQR 43.0–51.0)
Diastolic function	
E/A (*n* = 79)	
<1.5	85 (85.0 %)
≥1.5	15 (15.0 %)
E/e' septal (*n* = 72)	
<14	60 (83.3 %)
≥14	12 (16.7 %)
Right ventricular function	
TAPSE ≥ 14 mm (*n* = 77)	73 (94.8 %)
Pulmonary artery pressure (mmHg) (*n* = 51)	24.0 (IQR 15.0–31.0)
Moderate to severe valvular dysfunction (*n* = 87)	7 (8.0 %)
Pericardial effusion (*n* = 89)	4.5 (4.5 %)

*BMI, body mass index; vv-ECMO, venovenous extracorporeal membrane oxygenation; SOFA, Sequential Organ Failure Assessment; CRP, c-reactive protein; NT-proBNP, N-terminal pro-brain natriuretic peptide; hs-cTnT, high sensitive troponin T; LVEDD, left ventricular end diastolic diameter; DT, deceleration time; TAPSE, tricuspid annular plane systolic excursion; LVEF, left ventricular ejection fraction; MR, mitral regurgitation; AR, aortic regurgitation; TR, tricuspid regurgitation; ICU, intensive care unit*.

### Biomarkers

**Figure 2:**
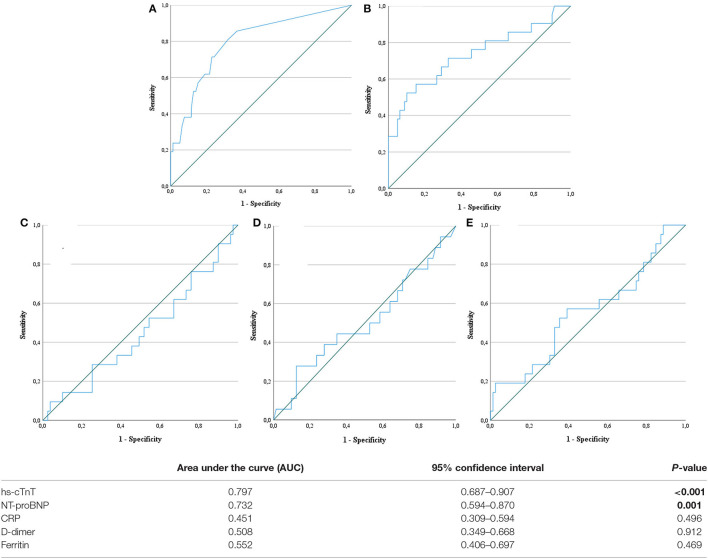
Receiver operating characteristic curve. Receiver operating characteristic (ROC) curves for five biochemical markers: high-sensitive troponin T **(A)**, N-terminal pro-brain natriuretic peptide **(B)**, C-reactive protein **(C)**, D-dimer **(D)** and ferritin **(E)**. hs-cTnT, high-sensitive troponin T; NT-proBNP, N-terminal pro-brain natriuretic peptide. Statistically significant result are marked in bold.

**Figure 3:**
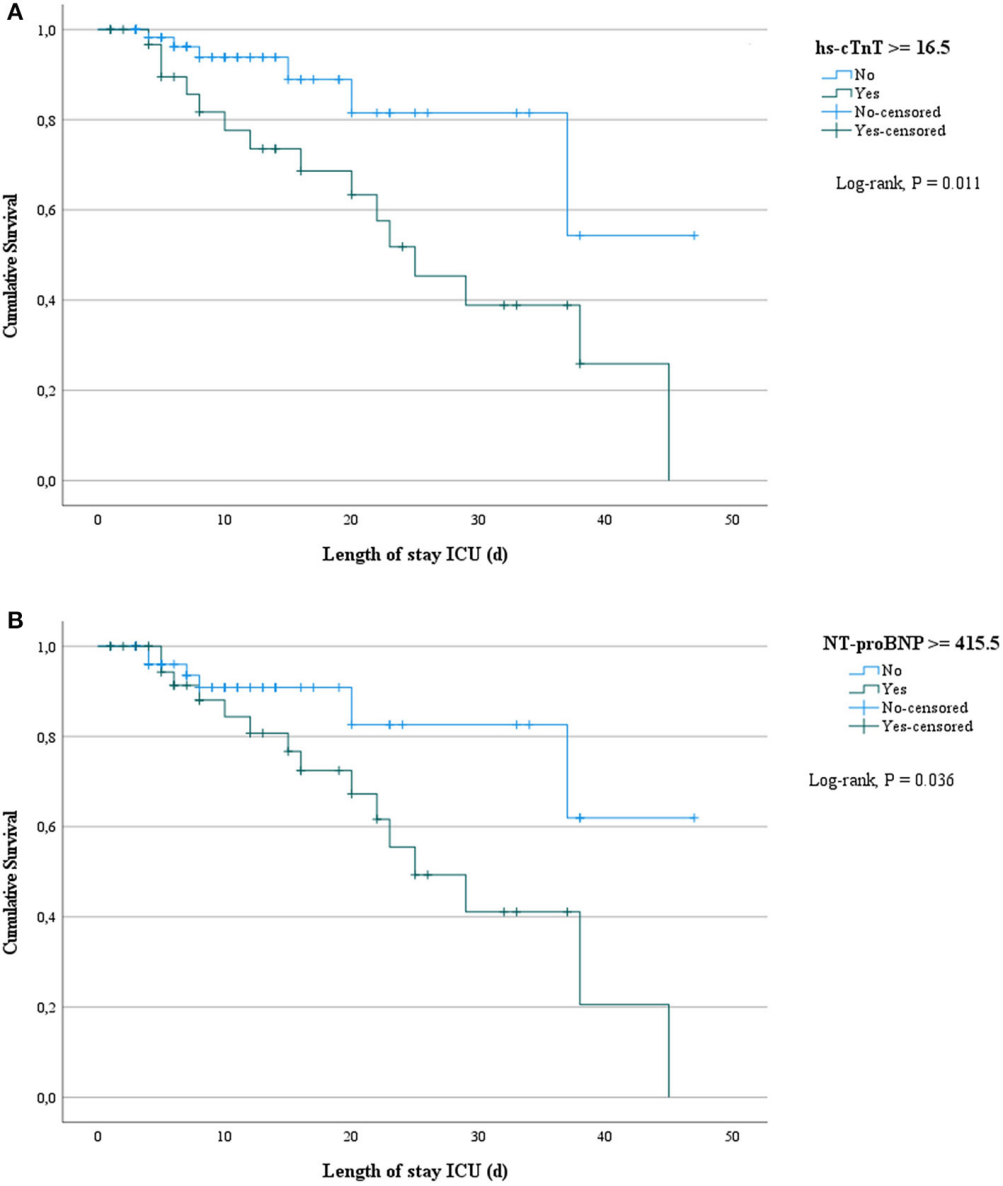
Survival analysis based upon the level of hs-cTnT and NT-proBNP on admission to ICU. Survival analysis based upon both levels of high sensitive troponin T **(A)** and N-terminal pro-brain natriuretic peptide **(B)** on admission to ICU. hs-cTnT, high-sensitive troponin T; NT-proBNP, N-terminal pro-brain natriuretic peptide.

**Figure 4:**
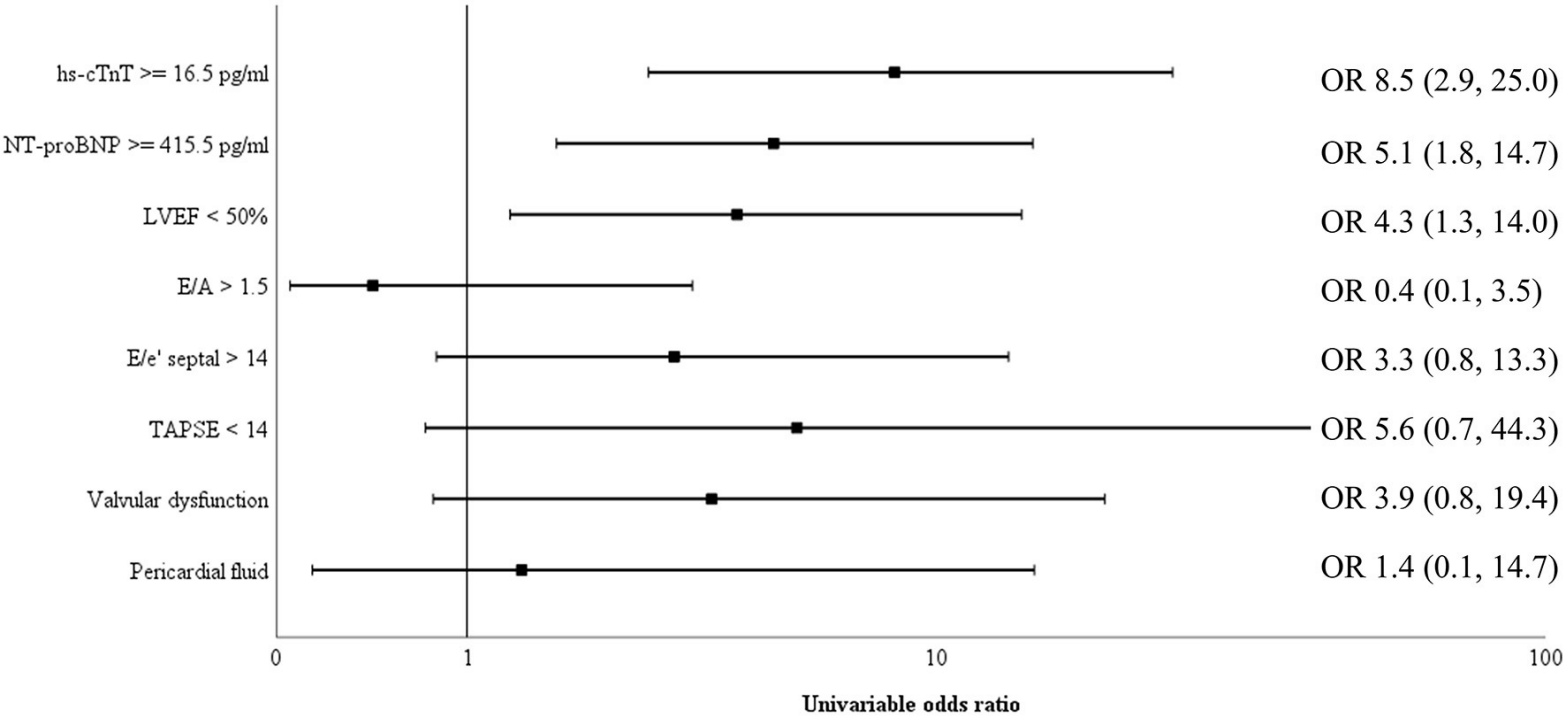
Univariable odds ratio for 30-day all-cause mortality. Univariable odds ratio for 30-day all-cause mortality for cardiac biomarkers hs-cTnT and NT-proBNP as well as several echocardiographic measurements (reduced left ventricular ejection fraction (LVEF), increased E/e', increased E/A, decreased tricuspid annular plane systolic excursion (TAPSE), valvular dysfunction and pericardial fluid). Both elevated cardiac biomarkers above their respective cut-off value and a reduced LVEF had a significant higher odds ratios for 30-day all-cause mortality. NT-proBNP, N-terminal pro-brain natriuretic peptide; hs-cTnT, high sensitive troponin T; TAPSE, tricuspid annular plane systolic excursion; LVEF, left ventricular ejection fraction; OR, odds ratio.

**Figure 5:**
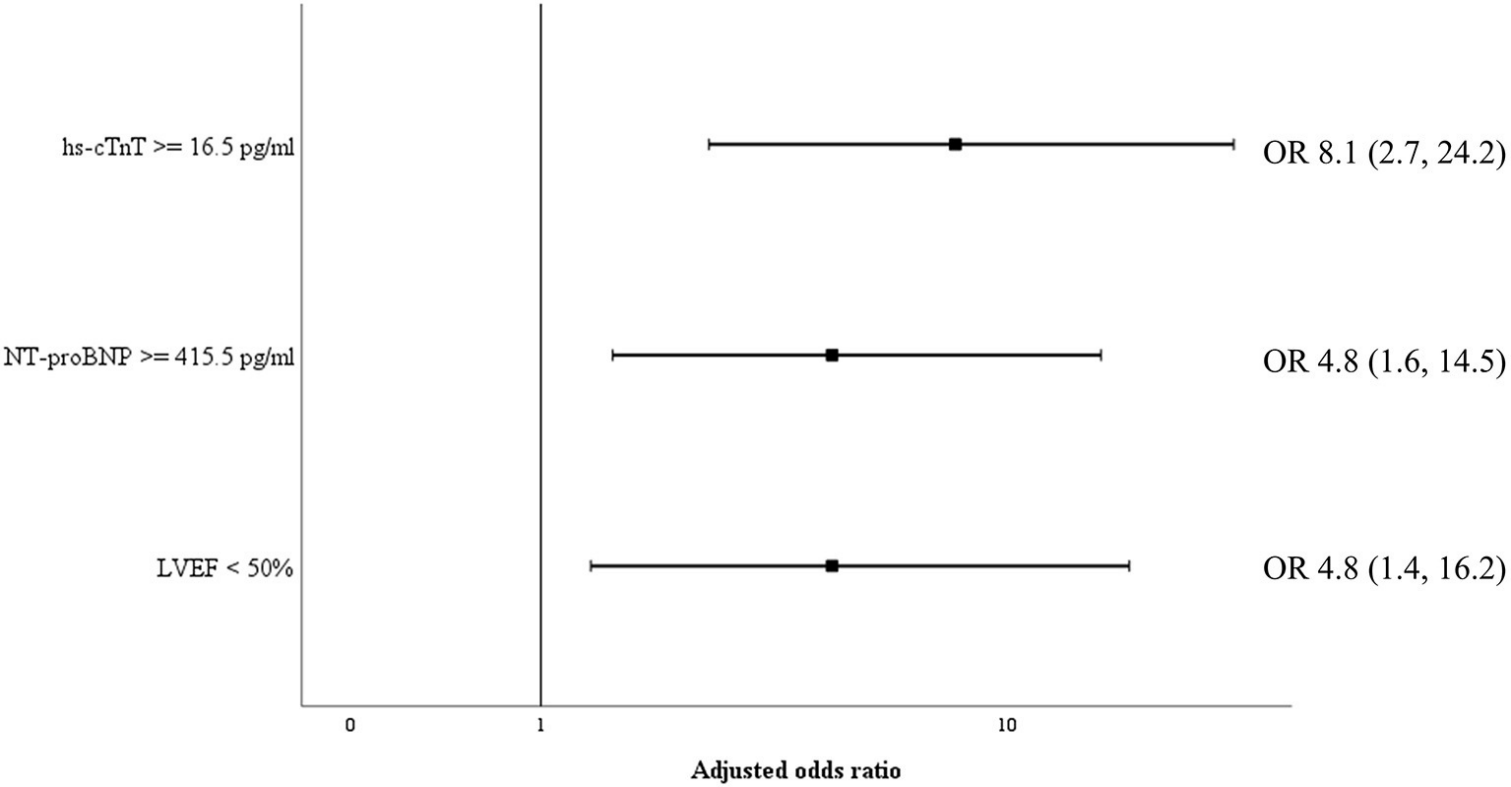
Adjusted odds ratio for 30-day all-cause mortality. Odds ratio for 30-day all-cause mortality, adjusted for SOFA-score, for cardiac biomarkers hs-cTnT and NT-proBNP as well as reduced left ventricular ejection fraction. NT-proBNP, N-terminal pro-brain natriuretic peptide; hs-cTnT, high sensitive troponin T. LVEF, left ventricular ejection fraction; OR, odds ratio.

Biomarkers of inflammation (CRP and ferritin) and D-dimer did not differ significantly among survivors and non-survivors. Cardiac biomarkers were elevated in almost half of all included patients: hs-cTnT ≥ 14 pg/ml in 47%, and NT-proBNP ≥ 450 pg/ml in 39%. The level of these biomarkers was significantly higher in non-survivors (Table 2). Figure 2 shows a ROC-curve for all 5 biomarkers with their respective area under the curve (AUC). CRP, ferritin and D-dimer were not associated with mortality, while the association of hs-cTnT (AUC: 0.79) and NT-proBNP (AUC: 0.71) was fair. Based on our data, we explored an optimal cut-off value for risk prediction for hs-cTnT and NT-proBNP. A value of 16.5 pg/ml for hs-cTnT corresponded with sensitivity and specificity for mortality of resp. 71.4 % and 48.6 %. The univariable odds ratio for 30-day all-cause mortality in patients with hs-cTnT ≥ 16.5 pg/ml was 8.5 (95% CI 2.9, 25.0). For NT-proBNP, an optimal cut-off value of 415.5 pg/ml corresponded with sensitivity and specificity for mortality of resp. 71.4 % and 38.5 %. The univariable odds ratio for 30-day all-cause mortality in patients with NT-proBNP ≥ 415.5 pg/ml was 5.1 (95% CI 1.8, 14.7). When adjusted for age, the adjusted odds ratio for 30-day all-cause mortality in patients with hs-cTnT ≥ 16.5 pg/ml was 7.1 (95% CI 2.3, 21.7; *P* = 0.001). For NT-proBNP a cut-off value of 415.5 pg/ml corresponded with an adjusted odds ratio of 3.5 (95% CI 1.1, 10.9; P 0.029). When adjusted for gender, the adjusted odds ratio for 30-day all-cause mortality in patients with hs-cTnT ≥ 16.5 pg/ml was 7.3 (95% CI 2.4, 21.9; *P* < 0.001). For NT-proBNP a cut-off value of 415.5 pg/ml corresponded with an adjusted odds ratio of 4.6 (95% CI 1.6, 13.4; *P* 0.006). When adjusted for kidney function (eGFR < or ≥ 60 mL/min), the adjusted odds ratio for 30-day all-cause mortality in patients with hs-cTnT ≥ 16.5 pg/ml was 8.1 (95% CI 2.7, 24.6; *P* < 0.001). For NT-proBNP a cut-off value of 415.5 pg/ml corresponded with an adjusted odds ratio of 4.5 (95% CI 1.5, 13.2; *P* 0.006). When adjusted for SOFA-score, the adjusted odds ratio for 30-day all-cause mortality in patients with hs-cTnT ≥ 16.5 pg/ml was 8.1 (95% CI 2.7, 24.2; *P* < 0.001). For NT-proBNP a cut-off value of 415.5 pg/ml corresponded with an adjusted odds ratio of 4.8 (95% CI 1.6, 14.5; *P* 0.005). A survival analysis based upon the level of hs-cTnT and NT-proBNP on admission to ICU is presented in Figure 3. The unadjusted and adjusted odds ratios for 30-day all-cause mortality are in Figures 4, 5.

**Table 2:** Distribution of baseline demographics, disease severity, laboratory assessments, and echocardiographic parameters of patients between survivors and non-survivors.

	**Survivors**	**Non-survivors**	***P*-value**
	**(*n* = 79)**	**(*n* = 21)**	
**Demographics**			
Age (y)	61.0 (IQR 52.0–71.0)	69.0 (IQR 66.5–72.0)	**0.008**
Sex			
Male	48 (60.8 %)	18 (85.7 %)	**0.032**
Female	31 (39.2 %)	3 (14.3 %)	
BMI (kg/m^2^)	28.9 (IQR 25.7–33.9)	25.8 (IQR 22.4–31.4)	**0.034**
Smoking history			0.338
No smoker	48 (60.8%)	9 (42.9%)	
Former smoker	26 (32.9%)	10 (47.6%)	
Active smoker	5 (6.3%)	2 (9.5%)	
Transferred from			0.826
Emergency department	36 (45.6%)	8 (38.1%)	
Pneumology ward	17 (21.5%)	5 (23.8%)	
Other hospital (not ICU)	26 (32.9%)	8 (38.1%)	
Length of stay ICU (d)	10.0 (IQR 5.0–16.0)	15.0 (IQR 6.5–24.0)	0.085
**Comorbidities**			
Arterial hypertension	33 (41.8 %)	9 (42.9 %)	0.929
Kidney disease upon admission			**0.031**
eGFR <30 mL/min	1 (1.3%)	2 (9.5%)	
eGFR 30–60 mL/min	11 (13.9%)	6 (28.6%)	
eGFR > 60 mL/min	67 (84.8%)	13 (61.9%)	
Diabetes mellitus	22 (27.8 %)	6 (28.6 %)	0.948
Obstructive sleep apnea	2 (2.5%)	4 (19.0%)	**0.005**
Chronic obstructive pulmonary disease / asthma	2 (2.5%)	6 (28.6%)	** <0.001**
Hypercholesterolemia	27 (34.2%)	13 (61.9%)	**0.021**
Coronary artery disease	12 (15.2%)	5 (23.8%)	0.350
Peripheral artery disease	3 (3.8%)	3 (14.3%)	0.072
**Chronic medication**			
Use of statins	25 (31.6%)	12 (57.1%)	**0.031**
Use of antidiabetic drugs			
Metformin	20 (25.3%)	4 (19.0%)	0.550
SGLT2 inhibitor	0 (0.0%)	2 (9.5%)	**0.006**
Other	11 (13.9%)	4 (19.0%)	0.559
Use of antihypertensive drugs			
ACE-inhibitor	22 (27.8%)	6 (28.6%)	0.948
Beta blocker	31 (39.2%)	9 (42.9%)	0.764
Other	22 (27.8%)	3 (14.3%)	0.202
Use of antithrombotic / anticoagulant drugs			
Aspirin	13 (16.5%)	7 (33.3%)	0.086
P_2_Y_12_ inhibitor	2 (2.5%)	2 (9.5%)	0.146
NOAC or VKA	7 (8.9%)	5 (23.8%)	0.061
**Medication administered on ICU**			
Dexamethasone	56 (70.9%)	18 (85.7%)	0.169
Remdesivir	6 (7.6%)	6 (28.6%)	**0.009**
Hydroxychlorquine	10 (12.7%)	1 (4.8%)	0.304
Convalescent plasma	8 (10.1%)	2 (9.5%)	0.935
**Severity of illness at time of inclusion**			
Use of vasopressors	36 (45.6 %)	18 (85.7 %)	**0.001**
Use of mechanical ventilation	42 (53.2 %)	18 (85.7 %)	**0.007**
Use of vv-ECMO	4 (5.1 %)	3 (14.3 %)	0.141
Total SOFA-score	3.0 (IQR 2.0–8.0)	4.0 (IQR 2.0–11.5)	0.342
Respiratory SOFA-score	2.0 (IQR 2.0–3.0)	2.0 (IQR 2.0–3.0)	0.784
P/F ratio (IQR)	96.3 (IQR 70.2–120.6)	92.9 (IQR 74.5–153.5)	0.375
**Inflammatory markers at time of inclusion**			
CRP (mg/L)	137.1 (IQR 69.0–208.0)	125.5 (IQR 56.5–200.5)	0.496
D-dimer (ng/mL)	1025.0 (IQR 640.0–1740.0)	965.0 (IQR 625.0–2885.0)	0.912
Ferritin (μg/L)	1079.0 (IQR 661.0–2271.0)	1492.0 (IQR 603.0–3072.0)	0.469
**Cardiac biomarkers at time of inclusion**			
hs-cTnT (μg/L)			
≥16.5 μg/L	18 (22.8 %)	15 (71.4 %)	**<** **0.001**
<16.5 μg/L	61 (77.2 %)	6 (28.6 %)	
NT-proBNP (pg/mL)			
≥415.5 pg/mL	26 (32.9 %)	15 (71.4 %)	**0.001**
<415.5 pg/mL	53 (67.1 %)	6 (28.6 %)	
**Echocardiography parameters at time of inclusion**			
LVEF (%)			
Normal (>50%)	62 (86.1 %)	10 (58.8 %)	**0.011**
Midrange (40–50%)	10 (13.9 %)	6 (35.3 %)	
Reduced (<40%)	0 (0.0 %)	1 (5.9 %)	
LVEDD (mm)	47.0 (IQR 43.0–51.0)	46.0 (IQR 40.0–54.0)	1.000
Diastolic function			
E/A	
<1.5	55 (83.3 %)	12 (92.3 %)	0.410
≥1.5	11 (16.7 %)	1 (7.7 %)	
E/e′ septal	
<14	52 (86.7 %)	8 (66.7 %)	0.090
≥14	8 (13.3 %)	4 (33.3 %)	
Right ventricular function			
TAPSE ≥ 14 mm	62 (96.9 %)	11 (84.6 %)	0.069
Pulmonary artery pressure (mmHg)	22.0 (IQR 11.8–30.0)	29.0 (IQR 26.0–37.0)	**0.043**
Moderate to severe valvular dysfunction	4 (5.6 %)	3 (18.8 %)	0.081
Pericardial effusion	3 (4.2 %)	1 (5.9 %)	0.759

*BMI, body mass index; vv-ECMO, venovenous extracorporeal membrane oxygenation; SOFA, Sequential Organ Failure Assessment; CRP, c-reactive protein; NT-proBNP, N-terminal pro-brain natriuretic peptide; hs-cTnT, high sensitive troponin T; LVEDD, left ventricular end diastolic diameter; TAPSE, tricuspid annular plane systolic excursion; LVEF, left ventricular ejection fraction; MR, mitral regurgitation; AR, aortic regurgitation; TR, tricuspid regurgitation; ICU, intensive care unit. Statistically significant result are marked in bold*.

### Echocardiography

Transthoracic echocardiography was not feasible in 11 patients (11%) due to poor visualization or prone ventilation. LVEF was reduced in 19.1% of patients (Table 1). One patient had a severely reduced LVEF of 25%, which was pre-existing due to a non-ischemic dilated cardiomyopathy. Sixteen patients (18.0%) had a mildly reduced LVEF. Of these, four patients (25%) were known with coronary artery disease and a pre-existing mildly reduced ejection fraction. For the other portion of patients there was no history of coronary artery disease and no previous echocardiography available. However, during admission none of the patients had evidence for acute ischemic signs on a continuous 3-lead ECG and daily 12-lead ECG. LVEF was significantly lower in those who ultimately died (Table 2). Levels of hs-cTnT and NT-proBNP were elevated in, respectively, 38.9 and 34.7% of patients with normal LVEF. Right ventricular function, evaluated by TAPSE, was normal (≥14 mm) in 94.8% of our cohort. After dichotomization between normal and abnormal TAPSE (≥ vs. <14 mm), patients with an abnormal RV function had higher mortality but this increase was not significant. There was no significant difference between survivors and non-survivors concerning diastolic function. The presence of moderate to severe valvular regurgitation (aortic, mitral, and tricuspid) or pericardial effusion did not differ significantly between the two groups. When adjusted for SOFA-score, the adjusted OR for 30-day all-cause mortality in patients with a reduced LVEF was 4.8 (95% CI 1.4, 16.2; *P* 0.011), which is represented in Figure 5. When adjusted for age, the adjusted OR for 30-day all-cause mortality in patients with a reduced LVEF was 3.7 (95% CI 1.1, 12.3; *P* 0.034). When adjusted for gender, the adjusted OR for 30-day all-cause mortality in patients with a reduced LVEF was 3.9 (95% CI 1.2, 12.9; *P* 0.026). Unadjusted OR's for 30-day all-cause mortality for all echocardiographic findings are shown in Figure 4.

## Discussion

This prospective study in critically ill COVID-19 patients has six important findings: (I) elevated levels of hs-cTnT and NT-proBNP upon admission are common and were found in, respectively, 47 and 39% of patients, (II) Elevated cardiac biomarkers are not necessarily linked to ventricular dysfunction as around 40% of patients with normal ejection fraction had either elevated levels of hs-cTnT and/or NT-proBNP, (III) Elevated levels of hs-cTnT, and to a lesser extent, NT-proBNP were associated with mortality, (IV) Serum levels of frequently used biomarkers (C-reactive protein, D-dimer and ferritin) and other clinical parameters of disease-severity (total SOFA, respiratory SOFA and P/F ratio) were not predictive for 30-day all-cause mortality, (V) Decreased LV function was associated with worse prognosis, whereas diastolic dysfunction and impaired RV function were not, (VI) cardiac ultrasound was not possible for various reasons in as much as 11% of this cohort of critical COVID-19 patients.

Whether cardiac biomarkers should be systematically measured as part of the workup for every hospitalized COVID-19 patient remains subject of debate. Currently, the European Society of Cardiology (ESC) and the American College of Cardiology (ACC) recommend against their routine use, while awaiting more evidence, as they warn for unnecessary diagnostic investigations, risk exposure and medical overuse (16, 28). Another reason to not currently recommend the routine use of cardiac biomarkers in prognostication is the belief that these markers would only be of limited incremental prognostic value to other markers of disease-severity (31). Recent research showed for example that higher D-dimer levels on admission to ICU seem to be independently associated with higher risk of death in critical COVID-19 (32). This, however, contrasts with the findings in our study and previous research. In an early report of 191 patients with COVID-19 in Wuhan, the univariable odds ratio for mortality when hs-cTnT was above the 99th percentile upper reference limit was 80.1 (95% CI, 10.3–620.4; *P* < 0.0001) regardless of underlying cardiovascular disease. This was higher than for all other biomarkers or scores tested, including D-dimer, ferritin and SOFA-score (33). Another study by Manocha et al. showed that hs-cTnT was the only independent predictor of mortality among the same five biomarkers (i.e., CRP, ferritin, D-dimer, NT-proBNP and hs-cTnT), whereas Shi et al. found statistical significance for both hs-cTnT and NT-proBNP (34, 35). Our results are in line with these findings and support the statement of Sandoval et al. that the use of cardiac biomarkers for prognostic purposes may help in risk-stratification (36). We furthermore agree that this should not necessarily lead to unnecessary diagnostic testing when it is accompanied by clear education about the goals and implications of potentially elevated biomarkers (36).

We observed a reduced left and right ventricular function in, respectively 17 and 5.2% of our patients. Previous large-scale research found similar results concerning reduced left ventricular function (20%), whereas right ventricular function was reduced in about 30% (23). Based on our data, reduced left ventricular systolic function was associated with mortality. However, right ventricular function, assessed with TAPSE, which only estimates longitudinal right ventricular function, was not. Due to the low number of patients with reduced right ventricular contractility one should interpret this finding with caution. In previous research, left- and right ventricular function, analyzed with strain measurements, were both correlated with poor outcome (23, 26, 37). Diastolic dysfunction, based upon E/A and E/e′ measurement, was not associated with higher odds for 30-day all-cause mortality. A prospective study of Szekely et al. showed similar results for E/A, though elevated E/e' in their cohort was associated with a higher hazard ratio for death. However, this result just narrowly met statistical significance (HR 1.08, 95% CI: 1.001, 1.2) (22). Overall, comparison of echocardiographic findings in COVID-19 subjects is difficult given the large heterogeneity in study populations and measurement approaches (37).

The fact that patients with elevated cardiac biomarkers did not necessarily have a reduced LVEF underlines the hypothesis that cardiac injury in COVID-19 may be due to a myriad of causes including direct myocardial injury of SARS-CoV-2 and indirect myocardial stress due to respiratory failure, thrombogenicity, sympathetic stimulation, cytokine release and endothelial dysfunction (31, 38–40). In recent research using cardiac magnetic resonance imaging (cMRI), COVID-19 patients with elevated hs-cTnT of unknown origin showed to have both ischemic and non-ischemic alterations on cMRI. However, in 31% of cases even with cMRI no cause could be found and most subjects had a normal LVEF (93%) (41). As such, elevated cardiac biomarkers may represent disease severity in a more complete way than routine echocardiography.

Moreover, routine echocardiography is not always possible in real-world practice due to practical (poor visualization and prone ventilation) or logistic problems, which limits its use even more. In the present cohort echocardiography was not feasible in about one tenth of patients. Furthermore, it exposes health care personnel to contagious risks and may be more time-consuming due to disinfection protocols. Taken together, the additive value of routine echocardiography on top of the measurement of cardiac biomarkers is questionable, even though reduced left ventricular function may predict worse outcome. This is in line with the ESC guidance, which currently recommends against performing echocardiography in COVID-19 patients, unless it is likely to alter the management strategy (16).

The current study has some important strengths. First, the study population was critically ill and prospectively evaluated, which contrasts with most studies evaluating all hospitalized patients retrospectively. Second, the combination of a prospective assessment of biomarkers and echocardiography in the same study population is rather unique. To our knowledge, only two smaller similar series were previously published (42, 43). In these studies, LV dysfunction was common in patients with elevated serum levels of hs-cTnT, though also present in 12% of patients without elevated levels of hs-cTnT (42, 43). However, possible relationships between the levels of cardiac biomarkers or echocardiographic findings and outcome parameters were not studied.

Five study limitations should also be addressed. First, no serial data of cardiac biomarkers were obtained, although this could be of interest as dynamic changes and/or peak values during admission may add additive value in prognostication (36, 44). Second, extrapolation of these results should be done with caution as this was a single-center study in critical COVID-19 patients and criteria for admission to ICU may differ between hospitals. For instance, COVID-19 patients with mono-organ failure requiring high flow nasal cannula, as well as patients with established do-not-resuscitate orders were admitted to dedicated mid-care units and thus not included in the present study. Third, our study has a relatively small sample size and results must be validated in larger cohorts. Fourth, echocardiographic evaluation of LVEF was performed using eye-balling methodology and no other more advanced imaging techniques were obtained. Finally, the extent of preexisting cardiovascular disease was largely unknown and therefore no difference could be made between established cardiovascular disease and new COVID-19 induced cardiovascular abnormalities.

## Conclusion

This study highlights the strong predictive value of the cardiac biomarkers hs-cTnT and NT-proBNP taken upon ICU admission in critically ill COVID-19 patients. They outperform other routinely used biomarkers, as well as clinical indices of disease severity in ICU in this specific cohort. Transthoracic echocardiography has several limitations and should therefore only be considered if it is likely to impact therapeutic management.

## Data Availability Statement

The raw data supporting the conclusions of this article will be made available by the authors, without undue reservation.

## Ethics Statement

The studies involving human participants were reviewed and approved by Ethics Committee, University Hospital Ghent C. Heymanslaan 10 9000 Ghent, Belgium Number: BC-07568 Date: April 1st, 2020. The patients/participants provided their written informed consent to participate in this study.

## Author Contributions

HS, SG, and EH conceived the principal idea and critically revised the manuscript. BZ, SD, HS, and SG performed the echocardiography. BZ and SD were major contributors in data analysis and writing the manuscript. EH was prime investigator of the project and was a major contributor in the data analysis. All authors contributed to the article and approved the submitted version.

## Conflict of Interest

The authors declare that the research was conducted in the absence of any commercial or financial relationships that could be construed as a potential conflict of interest.

## Publisher's Note

All claims expressed in this article are solely those of the authors and do not necessarily represent those of their affiliated organizations, or those of the publisher, the editors and the reviewers. Any product that may be evaluated in this article, or claim that may be made by its manufacturer, is not guaranteed or endorsed by the publisher.
